# Initial pH determines the morphological characteristics and secondary metabolite production in *Aspergillus terreus* and *Streptomyces rimosus* cocultures

**DOI:** 10.1007/s00203-024-04186-y

**Published:** 2024-11-01

**Authors:** Tomasz Boruta, Martyna Foryś, Weronika Pawlikowska, Grzegorz Englart, Marcin Bizukojć

**Affiliations:** Faculty of Process and Environmental Engineering, Department of Bioprocess Engineering, Lodz University of Technology, ul. Wólczańska 213, Łódź, 93-005 Poland

**Keywords:** *Aspergillus terreus*, Coculture, Secondary metabolites, *Streptomyces rimosus*

## Abstract

The influence of the initial pH on the morphology and secondary metabolite production in cocultures and axenic cultures of *Aspergillus terreus* and *Streptomyces rimosus* was investigated. The detected secondary metabolites (6 of bacterial and 4 of fungal origin) were not found in the cultures initiated at pH values less than or equal to 4.0. The highest mean levels of oxytetracycline were recorded in *S. rimosus* axenic culture at pH 5.0. Initiating the axenic culture at pH 5.9 led to visibly lower product levels, yet the presence of *A. terreus* reduced the negative effect of non-optimal pH and led to higher oxytetracycline titer than in the corresponding *S. rimosus* axenic culture. The cocultivation initiated at pH 5.0 or 5.9 triggered the formation of oxidized rimocidin. The products of *A. terreus* were absent in the cocultures. At pH 4.0, the striking morphological differences between the coculture and the axenic cultures were recorded.

## Introduction

The discovery and production of microbial secondary metabolites (SMs) involve a great number of microbiological methods that have been developed over nearly 100 years since penicillin was discovered (Baral et al. 2018; Fleming 1929; Keller 2019). While the toolbox of experimental and computational approaches continues to expand, the fundamental goals behind the biotechnology-oriented research on SMs remain unchanged, namely to induce the biosynthesis of potentially useful target metabolites, investigate their biological activity, and develop the bioprocesses which yield the desired molecules at economically feasible productivities and titers (Nielsen and Nielsen 2017; Ramírez-Rendon et al. 2022). The morphology and pH are among the factors that strongly influence the formation of SMs in the cells of filamentous microorganisms, i.e., actinomycetes and filamentous fungi (Meyer et al. 2021; Brakhage 2013). The pH level affects the solubility and transport of nutrients into the cell, exerts stimulatory or inhibitory effects on the SM biosynthetic pathways, and contributes to shaping the morphological characteristics of fungal mycelium (Papagianni 2004). Notably, the optimal morphological form (dispersed hyphae, clumps or pellets) and the recommended pH level need to be determined experimentally for each microorganism and the target SM (Veiter et al. 2018). With regard to the industrially important fungal genus *Aspergillus*, the formation of pellets is known to be initiated by the agglomeration of spores, a process associated with the pH-dependent electrostatic properties of spore surfaces (Wargenau et al. 2013). As pointed out by Papagianni (2004), the tendency to form pellets generally increases together with the increase of medium pH. Carlsen et al. (1996) studied the pH-dependent morphological development of *Aspergillus oryzae* mycelium and reported the formation of dispersed hyphae at pH values between 3.0 and 3.5, the mixed (i.e., involving both dispersed hyphae and pellets) morphology at pH between 4.0 and 5.0, and the strictly pelleted growth at pH values above 6.0. Furthermore, spore agglomerates were recorded at the pH values higher than 4, while at lower pH levels (from 2.5 to 3.5) only the freely dispersed spores were observed (Carlsen et al. 1996). In a study concerning *Rhizopus* sp., Nyman et al. (2013) demonstrated that the probability of pellet formation at pH 5 was higher than at pH 4 or 6. Zhou et al. (2000) noted the presence of *Rhizopus oryzae* pellets at the pH values between 2.60 and 3.36, and also at 5.59. The fungal species *Mucor circinelloides* was reported to display pellet development due to the pH increase from the value of 3 to 5.3 (Xia et al. 2011). In the case of actinomycetes, it was shown for *Streptomyces tendae* that the decrease in medium pH to the level of 4 leads to considerable increase of pellet size (Vecht-Lifshitz et al. 1990). Notably, all the aforementioned morphology-related transitions of filamentous microorganisms take place at the pH values not exceeding 6, i.e., under acidic conditions. Here, inspired by the previously reported results, we conducted the cultivations of a biotechnologically relevant filamentous fungal species of remarkably rich SM catalogue (Amr et al. 2023; Boruta and Bizukojć 2016; Guo and Wang 2014), namely *Aspergillus terreus* (the producer of lovastatin, a cholesterol-lowering drug), to describe its morphology and SM repertoire at pH values between 2 and 6, i.e., within the pH range expected to yield a diverse spectrum of morphological forms. For comparative purposes, the cultures of a model actinomycete (Pšeničnik et al. 2024) *Streptomyces rimosus* (the producer of oxytetracycline, a broad-spectrum antibiotic) were investigated in parallel with *A. terreus* cultures. To the best of our knowledge, the SM landscapes of *A. terreus* and *S. rimosus* have never been investigated in relation to the pH-dependent morphological diversity. In addition, the experimental scope was expanded to include the two-species coculture system involving *A. terreus* and *S. rimosus*, which was previously investigated in the context of secondary metabolite production (Boruta et al. 2021, 2024) but without addressing the influence of pH on the coculture outcomes. To date, the effect of initial pH level of the medium on the repertoire of SMs in submerged cocultures of filamentous microbial species has not been described in literature. It remains unknown whether the pH-related observations made with regard to axenic cultures could be used to predict the results of the corresponding coculture variants as far as the biosynthesis of SMs and morphology are concerned. While the conventional microbiological approaches relying on axenic cultures (i.e., the cultures involving only a single organism) are well-established, validated and relatively easy to control, the methods involving microbial cocultures are becoming increasingly relevant in the SM-related studies (Nai and Meyer 2018). Most importantly, they are effective in terms of awakening silent biosynthetic gene clusters in microbial strains through providing the conditions for stimulatory interactions among the cocultured cells (Arora et al. 2020). However, the bioprocess-related context is often overlooked in the SM-oriented works involving microbial cocultures and the focus is typically on the discovery of novel molecules rather than on the development and characterization of cultivation processes. It should be emphasized that the cocultures are associated with many process variables absent from axenic cultures, i.e., the ones that reflect the interactions between two distinct microorganisms of different nutritional and temperature preferences, growth rates, biosynthetic repertoires, and morphological characteristics (Diender et al. 2021). It has been demonstrated that the inoculation ratio, medium composition, and relative inoculation time contribute collectively to shaping the outcomes of cocultures in terms of biomass growth and biosynthetic capabilities (Goers et al. 2014; Kapoore et al. 2022). However, the pH-related aspects of SM production in filamentous cocultures are yet to be addressed.

The aim of the study was to characterize the morphological forms and the SM repertoire in submerged cocultures and axenic cultures of *A. terreus* and *S. rimosus* under acidic conditions.

## Materials and methods

### Microorganisms and sporulation conditions

The strains *Aspergillus terreus* ATCC 20542 and *Streptomyces rimosus* ATCC 10970 were used in the experimental work. The spores of *A. terreus* were obtained via 10-day cultivation on agar slants (malt extract, 20 g L^− 1^; casein peptone, 5 g L^− 1^; agar, 20 g L^− 1^). For the sporulation of *S. rimosus*, the bacterium was cultivated for 10 days on ISP Medium 2 (BD, United States). The agar slants were stored at 4 °C.

### Cultivation medium

The liquid medium of the following composition was used: glucose, 20 g L^− 1^; lactose, 20 g L^− 1^; yeast extract, 5 g L^− 1^; KH_2_PO_4_, 1.51 g L^− 1^; MgSO_4_ ∙ 7 H_2_O, 0.52 g L^− 1^; ZnSO_4_ ∙ 7 H_2_O, 1 mg L^− 1^; NaCl, 0.4 g L^− 1^; Fe(NO)_3_ ∙ 9 H_2_O, 2 mg L^− 1^; biotin, 0.04 mg L^− 1^; trace element solution, 1 mL L^− 1^. The composition of trace element solution was as follows: H_3_BO_3_, 65 mg L^− 1^; CuSO_4_ ∙ 5 H_2_O, 250 mg L^− 1^; Na_2_MoO_4_ ∙ 2 H_2_O, 50 mg L^−^^1^; MnSO_4_ ∙ 7 H_2_O, 43 mg L^− 1^.

The pH of the medium was adjusted to the levels of 2.0, 3.0, 4.0, 5.0, and 6.0 (dependent on the variant) prior to sterilization by using 1 M solutions of NaOH or HCl. After 15 min of autoclaving at 121 °C, the medium was cooled down to the temperature of cultivation (i.e., 28 °C) and aliquots were taken to measure the post-sterilization pH values, which were equal to 2.0, 3.0, 4.0, 5.0, and 5.9. Hence, the values reported in this work as the initial pH, equal to 2.0, 3.0, 4.0, 5.0 and 5.9 are understood as the pH levels of the cultivation media after sterilization. During the three independent experiments performed over the course of the study, the differences in pH values between the corresponding media variants after sterilization did not exceed ± 0.03.

### Cultivation conditions

The inoculation of production medium was performed by transferring spore suspensions of *A. terreus* and *S. rimosus* (10 mL for each strain) to 200 mL of sterile liquid medium. The spore suspension was prepared by removing the spores from agar slants with the use of sterile plastic pipette and suspending them in a portion of sterile medium. The number of spores of each microorganism was adjusted with the use of a hemocytometer to reach the final spore concentration in the production medium at the level of (1.0 ± 0.1) × 10^9^ spores per liter. The cultivation was carried out in the laboratory shaker Innova S44i (Eppendorf, Germany) at 28 °C and 120 min^− 1^. The cultures were propagated in 500 mL flat-bottom glass flasks. The time of cultivation was equal to 168 h.

### Analysis of secondary metabolites

The biomass was removed via filtration and the liquid samples were analyzed with the use of AQUITY-UPLC apparatus coupled with SYNAPT G2 high-resolution mass spectrometer (WATERS, USA). For chromatographic (reversed-phase) separation of secondary metabolites, the column BEH Shield RP18 (2.1 mm × 100 mm × 1.7 μm) was used and the following water: acetonitrile gradient was applied: 0 min, 100:0; 2.5 min, 80:20; 5.5 min, 70:30; 7.5 min, 60:40; and 14.0 min, 40:60. The eluents contained formic acid at the concentration of 0.1% (v/v). The parameters of the mass spectrometry analysis were as follows: sampling cone, 40 V; capillary voltage, 3 kV; extraction cone, 4 V; temperature of the source, 120 °C; temperature of desolvation, 200 °C; ionization: ESI^−^. For the putative identification of secondary metabolites The Natural Product Atlas (van Santen et al. 2022) was employed, under the condition that the difference between the observed and calculated *m/z* values was less than 0.01. The identities of mevinolinic acid, oxytetracycline, and butyrolactone I were confirmed with the use of analytical standards. The standards of mevinolinic acid and oxytetracycline were obtained from Sigma-Aldrich (USA) and the standard of butyrolactone I was purchased from Enzo Life Sciences (USA). The peak areas corresponding to the assigned [M − H]^−^ peaks were determined by using TargetLynx software (WATERS, USA). For mevinolinic acid and oxytetracycline, the concentration values (in mg/L) were determined with the use of analytical standards. For the remaining metabolites, the peak area values (in “counts”) were obtained based on the number of counts recorded by the detector at a specified retention time. The characterization of SM repertoire in *A. terreus* vs. *S. rimosus* cocultures and the details regarding the experimental *m/z* values were described previously (Boruta et al. 2021, 2024).

### Microscopic observations

The observations were carried out with the use of OLYMPUS BX53 light microscope, OLYMPUS DP27 camera, and the software OLYMPUS cellSens Dimension Desktop 1.16 (Olympus Corporation, Tokyo). The microscopic observations were performed by using two objectives. For smaller objects, such as the pellets of *S. rimosus*, the 4× objective was used (calibration X: 1.15 μm/pixel, calibration Y: 1.15 μm/pixel). Whenever the object was too large to be observed via the 4× objective, the 2× objective was employed (calibration X: 2.3 μm/pixel; calibration Y: 2.3 μm/pixel). Each microscopic image was presented with a scale bar.

### Statistical analysis

Three independent experiments were performed (*n* = 3). Each result was presented as mean value ± standard deviation. The two-sample t test with the significance level α = 0.05 was applied to determine whether the differences between the variants were statistically significant. All calculations were carried out with the use of OriginPro 2017 software (OriginLab Corporation, USA).

## Results

The shake flask cocultures and axenic cultures involving *A. terreus* and *S. rimosus* were initiated at 5 different pH levels (2.0, 3.0, 4.0, 5.0, and 5.9). The investigation of experimental variants encompassed the microscopic observations of filamentous morphology, the analysis of SM repertoire based on ultra-performance liquid chromatography coupled with mass spectrometry (UPLC-MS), and the measurement of final pH levels of the cultures.

### Morphology

Filamentous microorganisms in the submerged cultures evolve in the following hyphal micromorphological (i.e., to be observed with the use of microscope) structures. These are unbranched hyphae, branched hyphae, clumps and pellets. Clumps are formed from entangled hyphae but have no distinctive core. Their shape is irregular. Pellets are spherical hyphal structures with the distinctive core in their center (Metz and Kossen 1977; Bizukojć and Ledakowicz 2010).

**Fig. 1:**
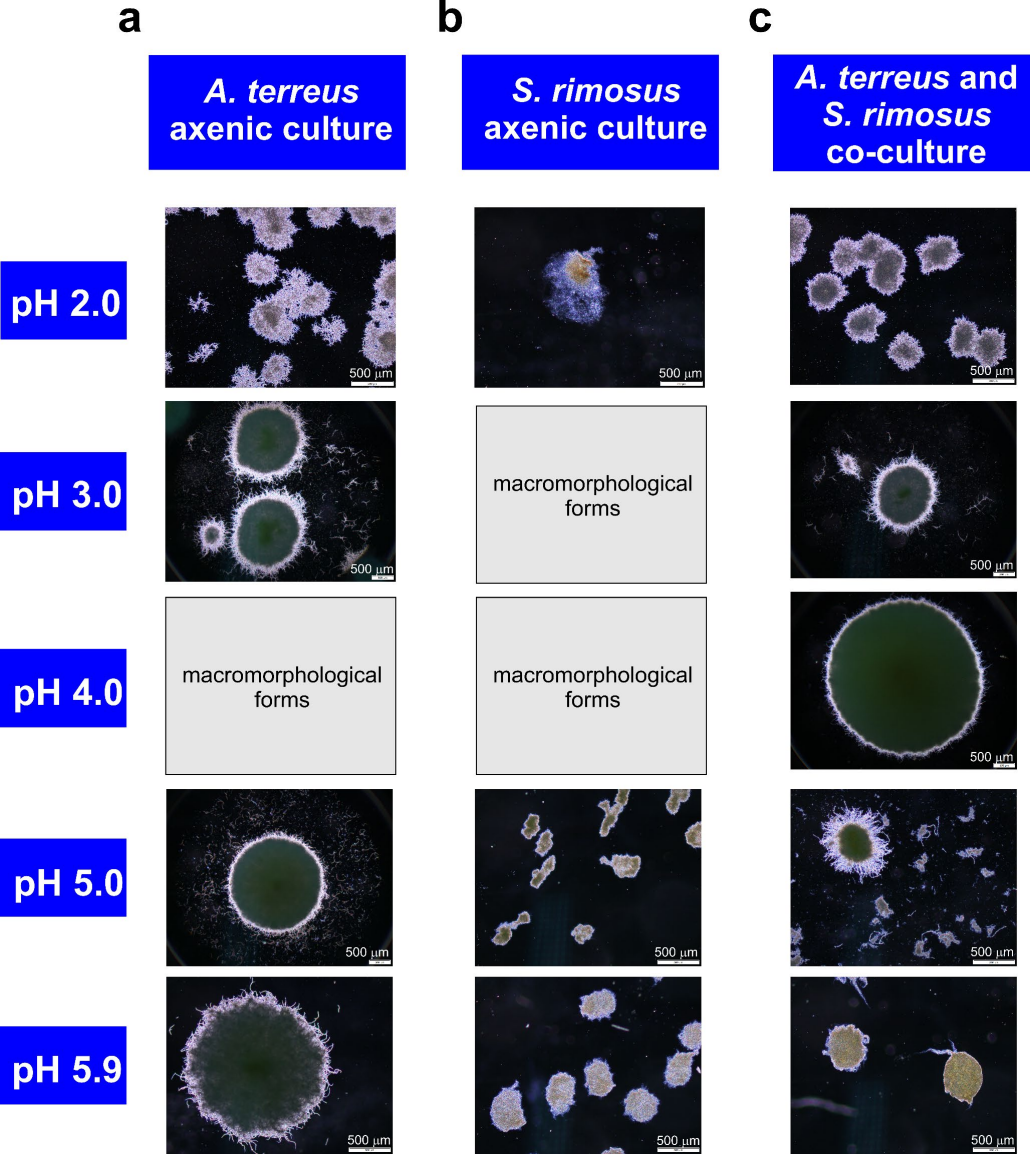
The microscopic images of morphological forms in *A. terreus* axenic culture (**a**), *S. rimosus* axenic culture (**b**), and *A. terreus* vs. *S. rimosus* coculture (**c**). The samples were drawn after 168 h of cultivation. The cultivations were performed in the medium containing glucose, lactose, yeast extract, KH_2_PO_4_, MgSO_4_ ∙ 7 H_2_O, ZnSO_4_ ∙ 7 H_2_O, NaCl, Fe(NO)_3_ ∙ 9 H_2_O, biotin, and trace element solution. The microorganisms were propagated in 500 mL flat-bottom glass flasks. The cultivation was carried out in the laboratory shaker at 28 °C and 120 min^− 1^. The macromorphological forms, too large to be observed by using the microscope, are not depicted here. Their photographic images are presented in Fig. 2

**Fig. 2:**
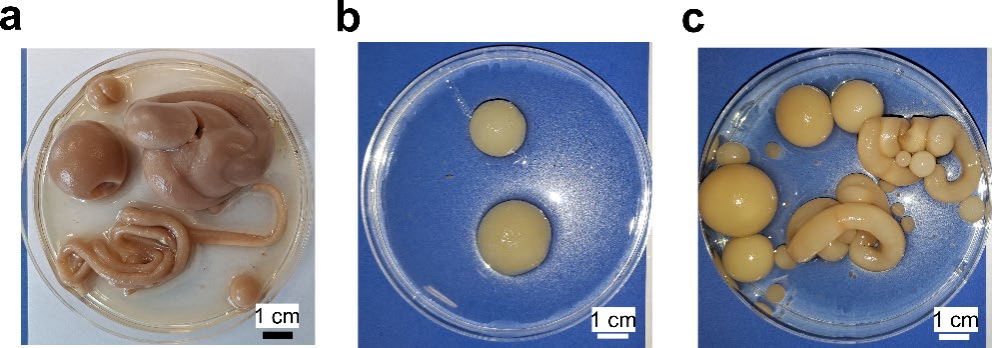
The photographic images of morphological forms in *A. terreus* axenic culture at the initial pH 4.0 (**a**), *S. rimosus* axenic culture at the initial pH 3.0 (**b**), and *S. rimosus* axenic culture at the initial pH 4.0

The morphological differences recorded among the investigated variants were striking (Figs. 1 and 2). At pH 2.0, *A. terreus* propagated in the form of moderately dense, hairy clumps with irregular boundaries formed by the radially growing filaments (Fig. 1a).These clumps fell apart from the spherical shape and lacked distinctive cores. Importantly, in this case one did not observe any formation of pellets with distinct cores that are normally formed due to spore agglomeration in *Aspergilli*, including *Aspergillus terreus* (Bizukojć and Ledakowicz 2010). Still, the loose branched and unbranched hyphae typically found at low pH values (Papagianni 2004) were not visible here. Markedly different behavior at the initial pH 2.0 was noted for *S. rimosus*, which displayed poor growth with only several irregular and barely visible biomass fragments found within the flask volume (Fig. 1b). These structures could have been developed due to slow biomass build-up on hyphal aggregates transferred from the agar slants during inoculation. In *A. terreus* vs. *S. rimosus* cocultures (Fig. 1c) the observed structures resembled the typical agglomerates of *A. terreus*, with dense and compact cores. Although not ideally spherical they were classified as pellets, as opposed to the dense clumps recorded in the *A. terreus* axenic cultures at pH 2.0. Arguably, the presence of *S. rimosus* spores promoted the formation of more typical *A. terreus*-like pellets in the coculture despite the low pH level. Hardly any traces of *S. rimosus* filaments were observed in the coculture.

At the initial pH 3.0, the formation of pellets containing a clearly visible core was recorded for *A. terreus* (Fig. 1a). Therefore, relative to the *A. terreus* culture initiated at pH 2.0, the morphological transition from clumps to pellets took place in the axenic *A. terreus* variant in response to the initial pH of 3.0. A drastic change with respect to pH 2.0 was also noted for *S. rimosus* axenic cultures, which at pH 3.0 displayed the growth in the form of macromorphological structures, i.e. large (bigger than 1 cm), smooth, olive-shaped biomass agglomerates (Fig. 2b). Only 2 or 3 structures of this type were formed within a single flask (210 mL), depending on the experimental replicate. As far as the coculture at pH 3.0 was concerned, the observed morphology closely resembled the one recorded in the axenic fungal counterpart, with the visible formation of *A. terreus*-like pellets (Fig. 1c).

Since the transition from clumped to pelleted growth took place in the pH 3.0 variant of *A. terreus* axenic culture, the formation of fungal pellets was expected to occur within the pH interval between 4.0 and 5.9. Surprisingly, at pH 4.0 a morphological peculiarity was recorded, manifested by the development of irregular, large, and structurally diverse biomass agglomerates of various shapes (Fig. 2a), which were too large to be observed by means of a microscope and thus classified as macromorphological forms. Similar morphological behavior was noted in the case of *S. rimosus* axenic variants again (Fig. 2c). However, the most interesting observation was made with regard to the coculture, which differed greatly from the axenic counterparts initiated at pH 4.0. Specifically, the co-cultivation of *A. terreus* and *S. rimosus* resulted in the formation of fungal-like pellets with dense cores and rather smooth, probably shaved, outer regions (Fig. 1c). Hence, the coculture which was started at pH 4.0 displayed the pelleted growth that could be monitored microscopically, as opposed to the corresponding axenic cultures of *A. terreus* and *S. rimosus*.

The pellets that were formed in the axenic cultures of *A. terreus* at pH 5.0 were similar to the ones observed at pH 3.0, albeit with less hairy outer regions (Fig. 1a). In the case of *S. rimosus*, the pH value of 5.0 was the lowest for which the pelleted growth occurred, however, the resulting pellets varied greatly in terms of shape, being rather irregular than spherical (Fig. 1b). Importantly, both the *A. terreus*-like and *S. rimosus*-like filamentous structures were clearly visible in the coculture at pH 5.0. One could notice the presence of larger pellets (resembling the pellets of *A. terreus*), the smaller pellets of various shapes (most probably representing *S. rimosus*), and the clumps and dispersed biomass of unknown microbial origin in the pH 5.0 coculture (Fig. 1c).

It was noted that the pellets of *A. terreus* (Fig. 1a) and *S. rimosus* (Fig. 1b) at pH 5.9 were larger than the ones found in the remaining axenic variants. Regarding the coculture at pH 5.9, solely the *S. rimosus*-like pellets were visible in the cultivation broth (Fig. 1c). Among the tested cocultures, only the variant propagated at pH 5.9 lacked any fungal-like filamentous structures.

To sum up, the cocultures initiated at pH 2.0, 3.0, and 4.0 revealed the presence of *A. terreus*-like pellets and no traces of morphological forms resembling *S. rimosus*. The opposite scenario (i.e., absence of *A. terreus*-like structures and the presence of *S. rimosus*-like forms) was recorded at pH 5.9. Finally, the microscopic images indicated that the coculture initiated at pH 5.0 promoted the simultaneous growth of *A. terreus* and *S. rimosus*.

### Production of secondary metabolites

**Fig. 3:**
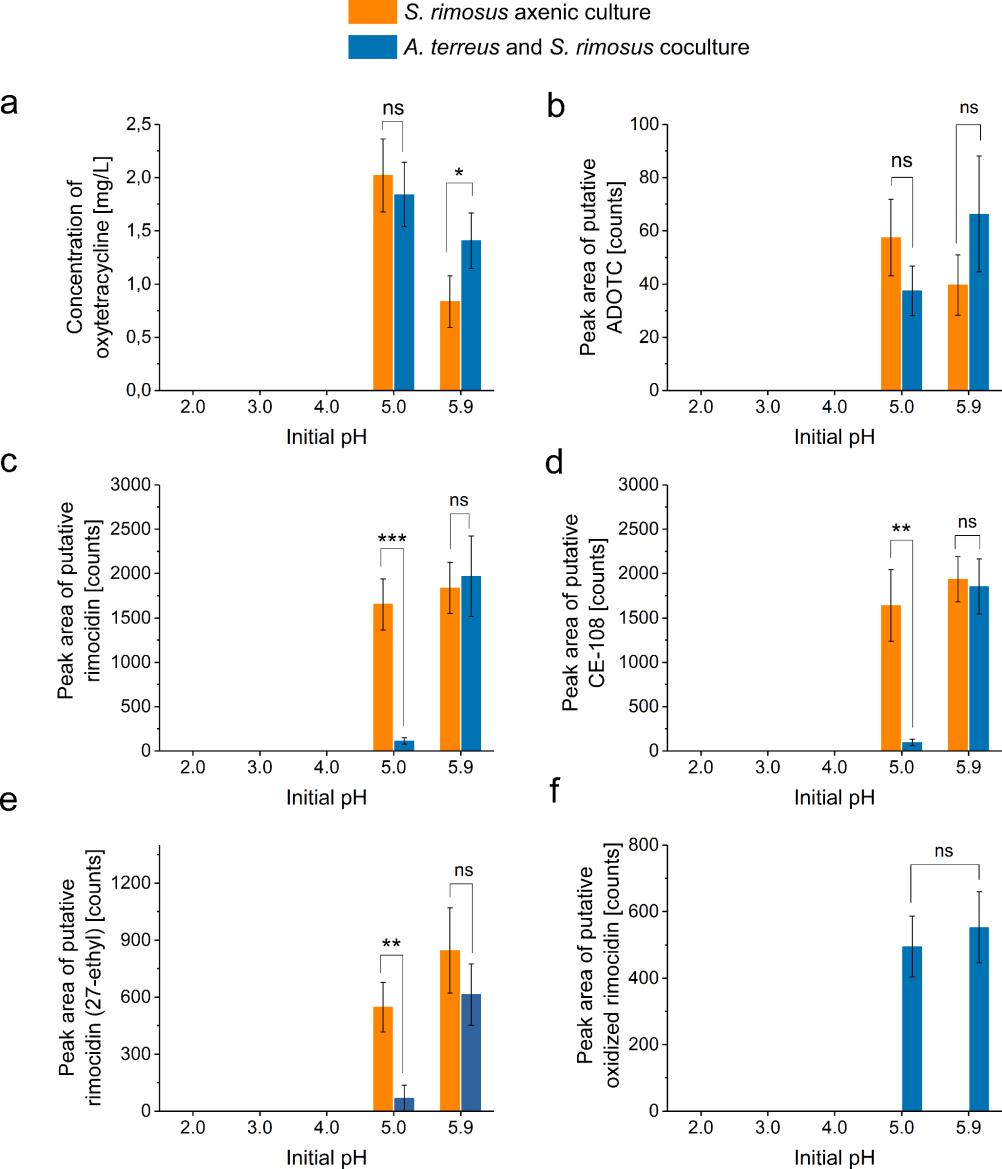
The levels of secondary metabolites produced in axenic cultures of *S. rimosus* and the cocultures of *A. terreus* and *S. rimosus* at different initial pH values, namely oxytetracycline (**a**), putative 2-acetyl-2-decarboxamido-oxytetracycline (ADOTC) (**b**), putative rimocidin (**c**), putative CE-108 (**d**), putative rimocidin (27-ethyl) (**e**) and putative oxidized rimocidin (**f**). The levels of secondary metabolites were determined after 168 h of cultivation. The secondary metabolites in *A. terreus* vs. *S. rimosus* cocultures were identified previously (Boruta et al. 2021, 2024). The results are given as the mean ± SD (*n* = 3). *, *P* < 0.05; **, *P* < 0.01; ***, *P* < 0.001; ns, not significant

The UPLC-MS analysis of cocultures revealed the presence of 6 SMs originating from *S. rimosus* and previously reported for the *A. terreus* vs. *S. rimosus* system (Boruta et al. 2021, 2024). This group of molecules included oxytetracycline, an antibiotic identified and assayed by using the analytical standard (Fig. 3a), and the closely related metabolite 2-acetyl-2-decarboxamido-oxytetracycline, abbreviated as ADOTC (Fig. 3b). In addition, 4 molecules putatively assigned to the rimocidin biosynthetic family (Boruta et al. 2021) were found in the coculture, namely rimocidin (Fig. 3c), CE-108 (Fig. 3d), rimocidin (27-ethyl) (Fig. 3e), and oxidized rimocidin, i.e., the metabolite resulting from the elimination of “CH_2_O_2_” moiety from rimocidin (Fig. 3f). Among the identified *S. rimosus* products, 5 molecules were found both in the cocultures and the axenic cultures (Fig. 3a-e), whereas one of the rimocidin derivatives, namely oxidized rimocidin, was detected exclusively under coculture conditions. Importantly, the production of all *S. rimosus* metabolites occurred only in the variants initiated at pH equal to 5.0 and 5.9 (Fig. 3). In other words, the SM biosynthetic activity was blocked at the pH values of 2.0, 3.0, and 4.0 regardless of the product or biosynthetic family. Considering the levels of SMs, no statistically significant differences between the cocultures and axenic cultures were observed for ADOTC (Fig. 3b), while for oxytetracycline (Fig. 3a) and the rimocidins (Fig. 3c, d, e) the outcome was clearly dependent on the initial pH level. With regard to the rimocidin family of molecules (Fig. 3c, d, e), their levels in axenic cultures at pH 5.0 were far higher than in the cocultures. By contrast, at pH 5.9 the production levels in cocultures and axenic variants did not exhibit significant differences (Fig. 3c, d, e). In the case of oxytetracycline, the modest production improvement (*P* = 0.048) was noted in the coculture relative to the axenic culture, but solely at pH 5.9 (Fig. 3a). At pH 5.0, the levels of oxytetracycline reached in coculture did not show significant differences compared with the axenic culture.

**Fig. 4:**
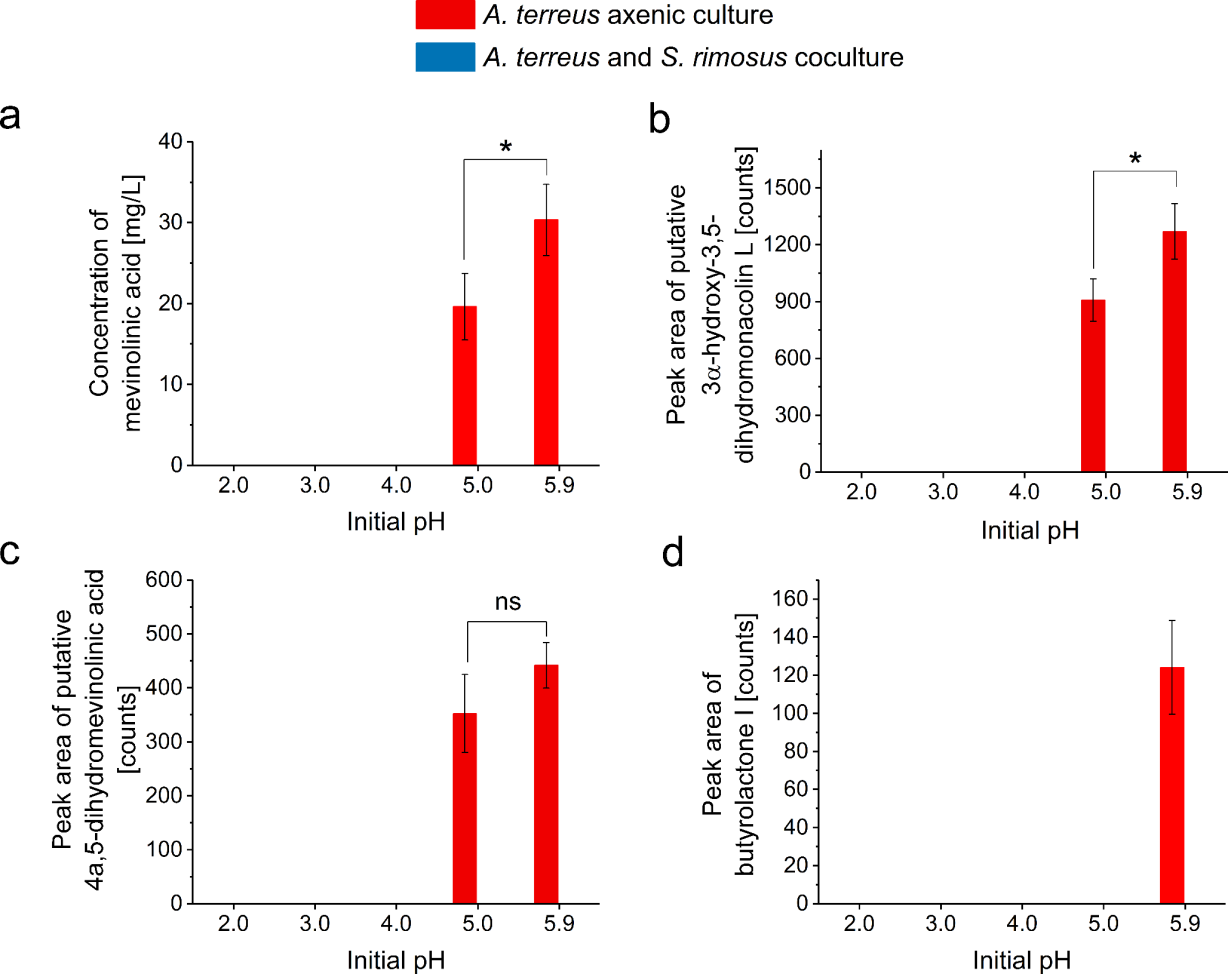
The levels of *A. terreus* secondary metabolites produced at different initial pH values, namely mevinolinic acid (**a**), putative 3α-hydroxy-3,5-dihydromonacolin L (**b**), putative 4a,5-dihydromevinolinic acid (**c**), and butyrolactone I (**d**). The levels of secondary metabolites were determined after 168 h of cultivation. The secondary metabolites in *A. terreus* vs. *S. rimosus* cocultures were identified previously (Boruta et al. 2021; 2024). The results are given as the mean ± SD (*n* = 3). *, *P* < 0.05; ns, not significant

Regarding the SMs produced by *A. terreus*, their presence was noted solely in the axenic variants (Fig. 4). This group included mevinolinic acid (i.e., lovastatin β-hydroxy acid), which was quantitatively assayed by using a standard solution (Fig. 4a), as well as the closely related and putatively identified molecules 3α-hydroxy-3,5-dihydromonacolin L (Fig. 4b) and 4a,5-dihydromevinolinic acid (Fig. 4c). These 3 metabolites, representing the statin biosynthetic pathway in *A. terreus*, were detected in the axenic cultures at pH 5.0 and 5.9. Another fungal SM identified in the broth, namely butyrolactone I, was found exclusively in the axenic culture at pH 5.9 (Fig. 4d). All the identified SMs of *A. terreus* were previously reported to be produced in *A. terreus* vs. *S. rimosus* cocultures (Boruta et al. 2021, 2024).

### Final pH

**Fig. 5:**
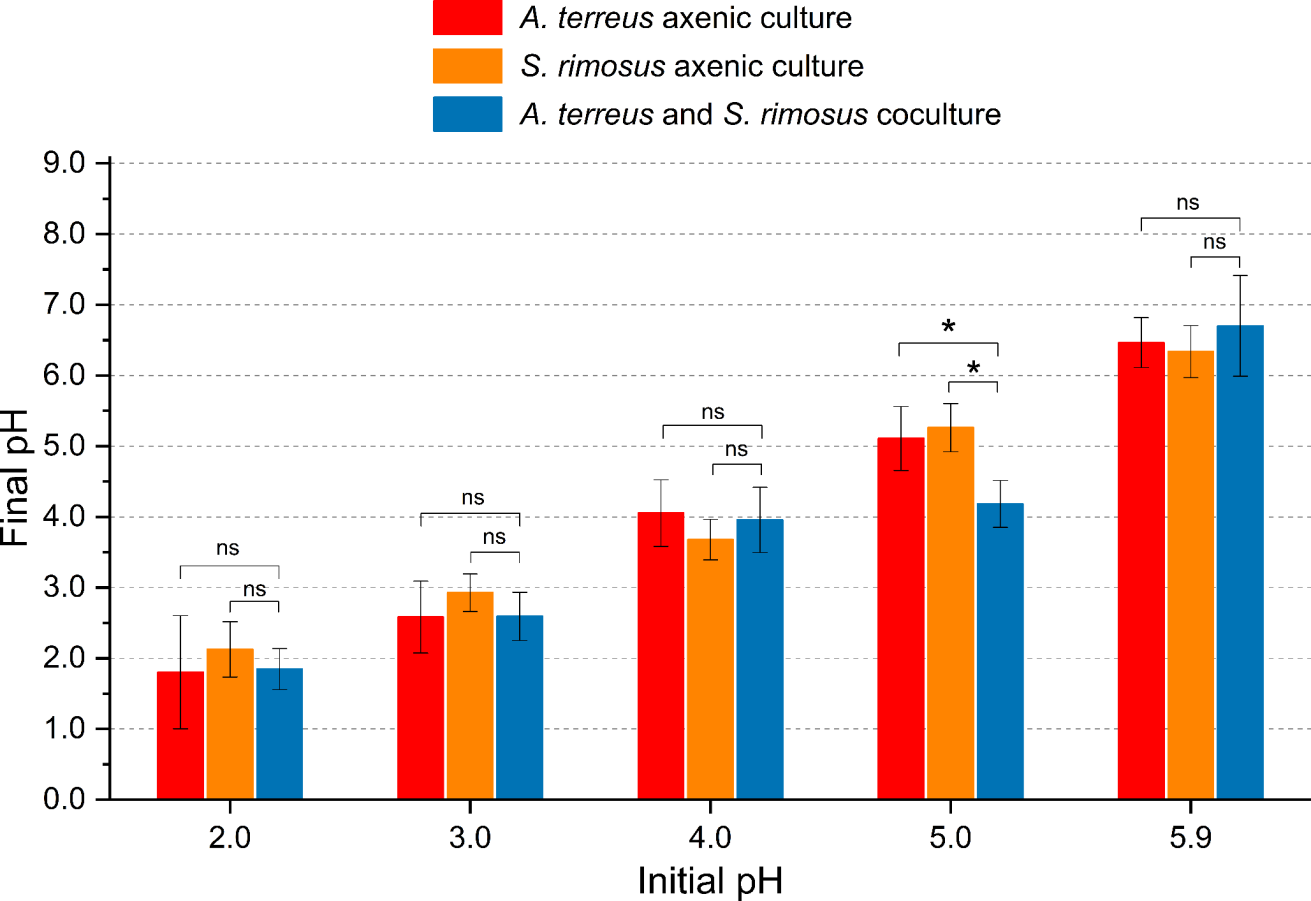
The final pH values (measured after 168 h of cultivation) in the *A. terreus* and *S. rimosus* axenic cultures and cocultures initiated at different pH values. The results are given as the mean ± SD (*n* = 3). *, *P* < 0.05; ns, not significant

Immediately after the completion of cultivation, the pH values were determined for the investigated cultures (Fig. 5). The statistically significant differences between the cocultures and the corresponding axenic cultures (*P* = 0.046 and 0.017 calculated with respect to *A. terreus* and *S. rimosus* axenic variants, respectively) were recorded only in the case of pH 5.0, with the mean final pH values equal to 5.11, 5.26 and 4.18 for the axenic culture of *A. terreus*, axenic culture of *S. rimosus* and the coculture, respectively. So, regarding the process which was started at pH 5.0, the non-negligible decrease in pH due to microbial cocultivation was observed compared to the axenic variants.

## Discussion

The influence of pH on the production of microbial SMs is associated with the pH preference of a given species in terms of biomass growth, and the biosynthetic pathway-specific effect of pH on the biosynthesis of a given SM (Brakhage 2013; Keller 2019). In cocultures, there are additional factors that contribute to the bioprocess outcome, e.g., the interspecies interactions and the differences among the cocultivated species with regard to their pH preferences. If the pH of the medium lies within the growth-promoting pH range of a given microorganism, the chances of survival of this microbe are higher than at less favorable pH levels. Of course, the pH is merely one of the factors that shape the outcome of microbial cocultivation, such as medium composition, inoculation time, inoculation ratio, and bioprocess conditions (Kapoore et al. 2022). Here, at pH levels 2.0, 3.0 and 4.0 the growth of *S. rimosus* biomass in the cocultures was not detectable, while the pellets closely resembling the morphological forms of *A. terreus* were developed (Fig. 1c). The opposite scenario was noted for the pH 5.9 variant, which resulted in the formation of *S. rimosus*-like pellets and no traces of *A. terreus* structures. Apparently, the pH 5.0 coculture was characterized by the parallel development of both species, yet the sizes of the *A. terreus*-like and *S. rimosus*-like morphological forms were smaller than in the axenic counterparts (Fig. 1a, b). Clearly, the lower pH values (i.e., 2.0, 3.0, and 4.0) favored the growth of fungal biomass at the cost of *S. rimosus*, while at pH 5.9 the actinomycete exerted its dominance and practically eliminated the fungus from the coculture. At pH 5.0, there was no clear dominance of any of the species, as they both coexisted in the coculture. Yet, the fungal SMs were not produced at pH 5.0 (Fig. 4) despite the presence of fungal-like morphological forms. Since the axenic variants of *A. terreus* at pH 5.0 did exhibit the production of SMs, the lack of fungal SMs in the coculture was evidently due to the presence of *S. rimosus*. As the growth of *A. terreus* was indeed observed in the coculture initiated at pH 5.0, the inhibition seemed to occur at the level of fungal secondary metabolic pathways rather than the growth-related primary pathways.

Most importantly, the effect of cocultivation of the production of a given SM (relative to the axenic culture) was shown to be dependent on the initial pH level. For example, the biosynthesis of oxytetracycline (Fig. 3a) was slightly enhanced in coculture relative to the axenic culture, however, this effect was recorded solely at pH 5.9. By contrast, for the coculture initiated at pH 5.0 the results were comparable with the levels noted for the axenic counterpart. In the case of rimocidin (Fig. 3c) and CE-108 (Fig. 3d), one could see their production being significantly inhibited due to cocultivation but only at pH 5.0. Based on these results, it is clear that the investigation of cocultivation-related effect on SM production need to be performed at various pH values to get a comprehensive view on this matter. In addition, one must be aware that the examination of pH effect on the SM production in axenic cultures does not necessarily reflect the correlations observed for the coculture counterparts.

To the best of our knowledge, this is the first report on the influence of pH on the morphological forms of *A. terreus* (Figs. 1a and 2a) and *S. rimosus* (Figs. 1b, 2b and c). The axenic growth of *S. rimosus* was barely visible at pH 2.0, occurred in the form of large irregular biomass agglomerates at pH 3.0 and 4.0, and, finally, manifested itself through the formation of pellets at pH 5.0 and 5.9. In the case of *A. terreus* axenic culture, there was a clumped and pelleted growth at pH 2.0 and 3.0, respectively. At pH 4.0, the micromorphological forms were replaced by macromorphological ones, i.e. large irregular biomass agglomerates. At higher pH values, i.e., 5.0 and 5.9, the pelleted growth was again observed. The morphological similarities and differences between *S. rimosus* and *A. terreus* were evident. Both species displayed the pelleted growth at pH 5.0 and 5.9 and the development of macromorphological biomass agglomerates at pH 4.0. The differences were, however, recorded at lower pH values. At pH 2.0, the growth of the actinomycete was poor (only the traces of biomass were visible in the flask), unlike the growth of *A. terreus*, which propagated in the form of clumps. At pH 3.0, *S. rimosus* formed macromorphological spherical biomass agglomerates (with the diameters exceeding 1 cm), whereas the fungus proliferated as pellets characterized by well-defined, distinct cores.

At pH 4.0, the coculture (Fig. 1c) differed markedly from its axenic counterparts (Fig. 2a, c) in terms of the displayed morphological characteristics. While the axenic cultures yielded large, macromorphological biomass agglomerates of various shapes and sizes, the growth in the coculture took the pelleted, *A. terreus*-like form. It is known that the agglomeration of *Aspergillus* spores strongly depends on culture pH, which in turn affects the spore surface charges (Grimm et al. 2005). Possibly, the formation of fungal-like pellets in the coculture may have taken place due to the charge shielding effect exhibited by *S. rimosus* spores on the conidia of *A. terreus*, what led to the agglomeration effect being distinct from the one observed in the fungal axenic culture. Presumably, the pH level of 4.0 favored the growth of *A. terreus* and its dominance over the actinomycete, what ultimately led to the development of pellets that resembled *A. terreus* rather than *S. rimosus* growth (Fig. 1c).

The coculture initiated at pH 5.0 was exceptional among the tested variants. Firstly, it was the only coculture in which both the *A. terreus*-like and *S. rimosus*-like morphological forms were recorded in the broth (Fig. 1c). Secondly, the final pH of this coculture was visibly lower than in the corresponding axenic cultures (Fig. 5). Thirdly, despite the presence of *A. terreus*-like pellets the coculture yielded no traces of *A. terreus* products (Fig. 4), what indicated the inhibition of fungal biosynthetic machinery due to *S. rimosus* dominance. Finally, it led to a marked inhibition in terms of the production of rimocidin and its derivatives compared with the *S. rimosus* axenic culture (Fig. 3c, d, e). At the same time, the coculture-related inhibition of oxytetracycline (Fig. 3a) and ADOTC (Fig. 3b) biosynthesis at pH 5.0 was less evident, with no statistically significant difference observed between the coculture and the axenic culture. In other words, at pH 5.0 the influence of cocultivation on the production of rimocidins was not comparable with the effect exerted on oxytetracycline and ADOTC formation, i.e., the response of SM biosynthetic route to cocultivation at a given pH varied among the pathways.

The study demonstrated that biomass growth alone is insufficient to predict SM production, regardless if the bioprocess is carried out under the axenic or coculture conditions. The biosynthesis of SMs in axenic cultures was recorded solely at pH 5.0 and 5.9, while the axenic growth of *A. terreus* and *S. rimosus* took place across the investigated pH range, with the only visible growth inhibition seen for *S. rimosus* at pH 2.0. In the cocultures, the presence of *A. terreus*-like morphological structures did not lead to fungal SM production, even if the *A. terreus*-like and *S. rimosus*-like forms coexisted (i.e., at pH 5.0). By contrast, the SMs of *S. rimosus* were detected in the broth whenever the *S. rimosus*-like pellets were formed in the coculture, i.e., at pH 5.0 and 5.9. Apparently, the effect of fungal growth at pH 5.0 was strong enough to decrease (but not eliminate) the rimocidin production by *S. rimosus* in the coculture, but it failed to block the actinomycete biomass development and oxytetracycline biosynthesis. At the same time, the growth of *S. rimosus* at pH 5.0 did not eliminate *A. terreus* biomass from the coculture, but it did block the production of detectable levels of all fungal SMs. So, the actinomycete seemed to exert a stronger SM production-blocking effect on the fungus than vice versa. As already mentioned, achieving the coexistence of two species in the coculture is clearly insufficient to ensure the SM production by any of them.

The lack of visible *A. terreus*-like pellets in the coculture initiated at pH 5.9 did not mean that the fungus had no influence on the coculture outcome, as indicated by the presence of the oxidized rimocidin, which was recorded exclusively under cocultivation conditions. It should be mentioned that this product was also seen in the coculture initiated at pH 5.0, a variant in which the fungal-like pellets were clearly observable. It means that the “*A. terreus* vs. *S. rimosus*” cocultures which were started at pH 5.0 or 5.9 yielded the rimocidin derivative regardless if the *A. terreus*-like pellets were formed (as in the “pH 5.0” coculture variant) or not (as in the pH “5.9” variant). Perhaps the spores of *A. terreus* were engulfed within the pellets of *S. rimosus* at pH 5.9, and the fungus still managed to exert the effect on the actinomycete from within the pellets. The origin of the rimocidin derivative could be associated with the biotransformation activity of the fungus, as previously suggested (Boruta et al. 2021), but its de novo biosynthesis by *S. rimosus* in response to the stimulus provided by *A. terreus* cannot be excluded.

In a previous study, Wargenau et al. (2011) investigated the pH-dependent electrostatic surface potential of *Aspergillus niger* conidia under acidic conditions. It was proposed that the surface potential depends on the release of melanin and the addition of negative charges to the outer layer of the spores. Importantly, the results indicated that the melanin release occurring under acidic pH is associated with the deprotonation of carboxyl groups bound to the pigment layer of the spores. The authors concluded that the pH-dependent repulsion between fungal spores depends on a number of factors, including the ionic strength of the medium, the thickness of surface coating, and the properties of individual ions present in the system. Since the events of spore agglomeration result from the net effect of repulsion and attraction between the spores, the tendency of a given species to form pellets cannot be attributed solely to the pH level. In the present study, the co-inoculation of *A. terreus* and *S. rimosus* spores at pH 4.0 resulted in the morphological outcome (i.e., the fungal-like pellet formation) that did not resemble the macromorphological structures recorded in the corresponding axenic variants. So, the presence of *S. rimosus* spores at pH 4.0 altered the agglomeration of *A. terreus* conidia, leading to the formation of pellets which were not seen in the corresponding axenic cultures. As already mentioned in the discussion, the relatively low pH apparently favored the growth of *A. terreus* at the cost of *S. rimosus*, so the observed macromorphological outcomes in the coculture initiated at pH 4.0 was the formation of *A. terreus*-like (not *S. rimosus*-like) pellets.

In the previous study, Bizukojć et al. (2012) demonstrated that relatively small amounts of mevinolinic acid can be found in the *A. terreus* axenic cultures initiated at pH values even as low as 3.5 and 4.5. In the present work, there was no evidence of mevinolinic acid presence in the variants initiated at pH less than 5.0. The discrepancy can be attributed to a large number of bioprocess-related differences between the two studies. Here, the composition of the growth medium included glucose and lactose as the sources of carbon, while glucose was absent from the medium used by Bizukojć et al. (2012). The previous study was based on the use of 24-h precultures, while the variants investigated in the present work were inoculated by applying the spore suspensions. In addition, the speed of the laboratory shaker, the cultivation temperature, the duration of the bioprocess, and the culture working volume used here differed from the ones reported by Bizukojć et al. (2012). All these factors can be expected to collectively contribute to SM production capabilities.

Finally, it must be remembered that the SM production (similarly as the morphological development) is associated with the net effect of numerous factors, not merely the pH level. It means that the medium composition, agitation, bioprocess scale, spore concentration, inoculation ratio, inoculum type (spores or preculture), relative inoculation time, etc., all contribute to the final outcome of microbial cocultivation. While the bioprocess-related aspects of SM production in axenic cultures represent a remarkably complex and demanding topic, the investigation of cocultures is even more challenging and complicated due to a large number of process variables associated with the interspecies interactions (Bertrand et al. 2014; Selegato and Castro-Gamboa 2023).

In conclusion, the effect of cocultivation on the morphological characteristics and the production of a given SM depends on the pH value of the medium. Specifically, the *A. terreus* vs. *S. rimosus* coculture initiated at the pH value of 5.9 leads to the elevated levels of oxytetracycline compared with the axenic variant. In addition, the coculture initiated at pH 5.0 results in the markedly decreased levels of rimocidin relative to the *S. rimosus* axenic culture. The molecule putatively identified as oxidized rimocidin is formed exclusively under coculture conditions. The coculture morphologies at pH 2.0, 3.0 and 4.0 resemble the pellets of *A. terreus*, whereas in the coculture initiated at pH 5.9 the *S. rimosus*-like pellets are formed. At pH 5.0, the coexistence of *A. terreus*-like and *S. rimosus*-like pellets takes place. At pH 4.0, the marked morphological differences between the coculture and axenic cultures are observed.

## Data Availability

No datasets were generated or analysed during the current study.
